# Cathodoluminescence as a probe of the optical properties of resonant apertures in a metallic film

**DOI:** 10.3762/bjnano.9.140

**Published:** 2018-05-18

**Authors:** Kalpana Singh, Evgeniy Panchenko, Babak Nasr, Amelia Liu, Lukas Wesemann, Timothy J Davis, Ann Roberts

**Affiliations:** 1 School of Physics, University of Melbourne, VIC 3010, Australia; 2 Centre for Neural Engineering, The University of Melbourne, VIC 3010, Australia; 3 Department of Electrical and Electronic Engineering, The University of Melbourne, VIC 3010, Australia; 4 Australian Research Council Centre of Excellence for Integrative Brain Function, The University of Melbourne, VIC 3010, Australia,; 5 Monash Centre for Electron Microscopy and School of Physics and Astronomy, Monash University, Clayton, VIC 3800, Australia

**Keywords:** cathodoluminescence, plasmonics

## Abstract

Here we present the results of an investigation of resonances of azimuthal trimer arrangements of rectangular slots in a gold film on a glass substrate using cathodoluminescence (CL) as a probe. The variation in the CL signal collected from specific locations on the sample as a function of wavelength and the spatial dependence of emission into different wavelength bands provides considerable insight into the resonant modes, particularly sub-radiant modes, of these apertures. By comparing our experimental results with electromagnetic simulations we are able to identify a Fabry–Pérot mode of these cavities as well as resonances associated with the excitation of surface plasmon polaritons on the air–gold boundary. We obtain evidence for the excitation of dark (also known as sub-radiant) modes of apertures and aperture ensembles.

## Introduction

The study of the interaction of electromagnetic waves with apertures in metallic films has been the subject of ongoing research following early investigations motivated by advances in radar and microwave technologies. In 1944 Bethe studied diffraction by small circular apertures in an infinitesimally thin, perfectly conducting film [1]. According to this theory, the transmission through an aperture in the limit where the wavelength (λ) is much smaller than the radius *b* (*b* << λ) varies as (λ/*b*)^4^. Subsequently, Bouwkamp extended Bethe's result adding further terms in a series expansion [2] and Roberts [3] developed a modal method to accommodate apertures in finite thickness films. A broad range of other apertures have been investigated including rectangular slots [4–5] and circular apertures [3,6] as well as more complex shapes such as cross-shaped [7–9], and coaxial apertures [10–11]. Interest in sub-wavelength metallic apertures increased significantly after Ebbesen et al. reported optical transmission, enhanced relative to an equivalent hole–area fraction of randomly arranged apertures, through periodic arrangements of holes in a silver film [12]. This enhancement is strongly associated with the ordered arrangement of the apertures. Theoretical and experimental research into isolated rectangular slots [4–5] and coaxial apertures [10–11], however, has shown that these cavities exhibit distinct localized resonances that have a strong dependence on the geometry of the holes including the thickness of the metal film [13]. Other than the excitations discussed above, sharp Fano resonances arising from the interference of two modes [14] have promising applications in sensing, switching and lasing. These resonances have been investigated in various structures such as thin film nanogratings [15], plasmonic oligomers [16], dolmen arrangements of nanorods [17] and ring–disk dimers [18]. Fano resonances have also been observed in nanoholes such as coaxial apertures [19] and dolmen nanocavities [18]. The performance of an array of double split-ring cavities [20] as biosensors using Fano resonances in the terahertz regime has been demonstrated. As is the case with nanoparticles, nanoholes play an important role as basic building blocks in a range of nanophotonic devices, including colour filters [21–24] and compact polarizers [25], exploiting resonant properties of the subwavelength apertures. The high sensitivity of the resonant modes of the apertures to the refractive index of the surrounding media underpins significant potential in realizing highly efficient ultra-compact biological and chemical sensors [26–31], plasmonic electrochemical sensors [32] and as SERS sensors [33]. Furthermore, by varying the geometry of the apertures across a surface, it is possible to introduce specific amplitude and phase profiles to the transmitted optical field [27]. This underpins the development of ultra-compact, planar, alternatives to conventional lenses [34]. Far-field optical investigations of isolated holes are, however, challenging since the throughput and reflectance are low despite the enhanced localized fields. Furthermore, plasmonic cavities exhibit a wide range of modes, many of which are “dark” to normally incident plane waves and challenging to excite using other optical methods. These modes are of intrinsic interest, however, and have also attracted attention due to their relatively high quality factor and long lifetimes that may underpin new optical sensors with a higher sensitivity and figure-of-merit than devices utilising “bright” dipole modes [35] and enhanced coupling to plasmonic cavities by emitters such as quantum dots [36]. One way to enhance the signal-to-noise ratio of transmission measurements is to study the collective optical properties of an array of apertures, choosing the periodicity to minimize both the effects of diffraction and coupling between unit cells [7]. Near field optical microscopy is another method that has been used to probe optical resonances of various nanostructures. Although the technique provides better-than-diffraction-limited resolution, image interpretation is complex due to the interaction between the tip and the sample. Nevertheless, progress has been made into the use of scanning probe methods for analysing modes of optical antennas [37].

Electron microscopy systems can also be used to probe various modes of optical nanostructures. Electrons in motion are accompanied by an electric field that varies in space and time [38], and hence, an electron beam can induce a time-varying polarization in an adjacent material leading to optical excitation. Optical resonances can be probed by studying either the loss in the energy of the electrons (through electron energy loss spectroscopy, EELS) [38–39] or the radiation emitted in the visible part of the electromagnetic spectrum through cathodoluminescence (CL) [38]. The relationship between the information obtained using EELS and CL has been studied theoretically [40]. CL is an established technique which is widely used in various fields to characterize a range of inorganic compounds such as ceramics, minerals and semiconductors [41–43]. CL has also been shown to be an invaluable tool in the investigation of optical modes of nanostructures [44]. These include characterising the plasmonic modes of silver nanoparticles [45] and resonant modes of single GaAs nanowires [46], modes of single and pairs of AlGaAs disks [47], the optical properties of quantum discs of GaN/AlN in GaN nanowires [48] and various modes of gold nanodecahedra [49]. The potential of CL used in transmission to observe various colour centres in nanodiamonds has also been demonstrated [50]. The spectral properties of core–shell CdSe/CdS quantum dots have also been studied using CL in a transmission electron microscope [51]. The same technique has been used to generate single photons and to characterize quantum states and the nature of the emitted beam with subwavelength resolution [52]. Dichroic-sensitive cathodoluminescence imaging has also been used to study the chiral nature of the gold split-ring resonators on a TiO_2_ substrate [53]. Most studies have focused on nanoparticles on silicon substrates that can have a significant impact on the optical resonances of plasmonic and other nanophotonic systems. Despite the significant body of work looking at the use of CL in characterising nanoparticles, there has been less attention directed at complementary nanoholes in metallic films. Coenen and Polman [54] investigated the properties of simple circular apertures in a 80 nm thick gold film on a silicon substrate. They showed that there was excitation of a magnetic mode of the aperture when excited near the edge of the aperture. Van de Haar et al. [55] also investigated a metamaterial consisting of a periodic array of metal–insulator–metal coaxial waveguides filled with Si and the subradiant, whispering gallery modes of circular grooves of various depths milled into a gold film have also been previously studied [56–57]. Symmetric and anti-symmetric modes of nanopore pairs in thin AlN/Au/AlN films [58] have been investigated using CL. Through the use of colloidal lithography, the authors [58] of were able to efficiently investigate a range of hole separations, but were restricted to weakly resonant circular apertures and pairs of holes.

Here we investigate the resonances of small groups of apertures in gold films on a glass substrate. Specifically, we study ensembles of three slot apertures using CL. It is well-known that simple slots in a metal film exhibit resonances, but there is a growing interest in the excitation of apertures complementary to resonant particles. Here we investigate ensembles of three simple slots arranged in the form of a triangle. This structure is complementary to a trimer consisting of three rods with the lowest energy magnetic dipole mode being dark and the next highest energy modes being degenerate orthogonal dipole modes (see Supporting Information File 1). The dominant modes of this structure are a dark mode with radial symmetry where the electric field in each aperture is radially directed from the centroid of the configuration and another, almost degenerate, radiant mode with a net dipole moment (see Supporting Information File 1). In both cases the electric fields in each aperture are similar to the dipole mode of a single slot, but the relative phase between the modes differs between the excitations in each aperture. Insight into our results is obtained through modelling the radiation emitted by a point dipole in close proximity to the air–gold surface using the finite element method.

## Experimental

**Figure 1:**
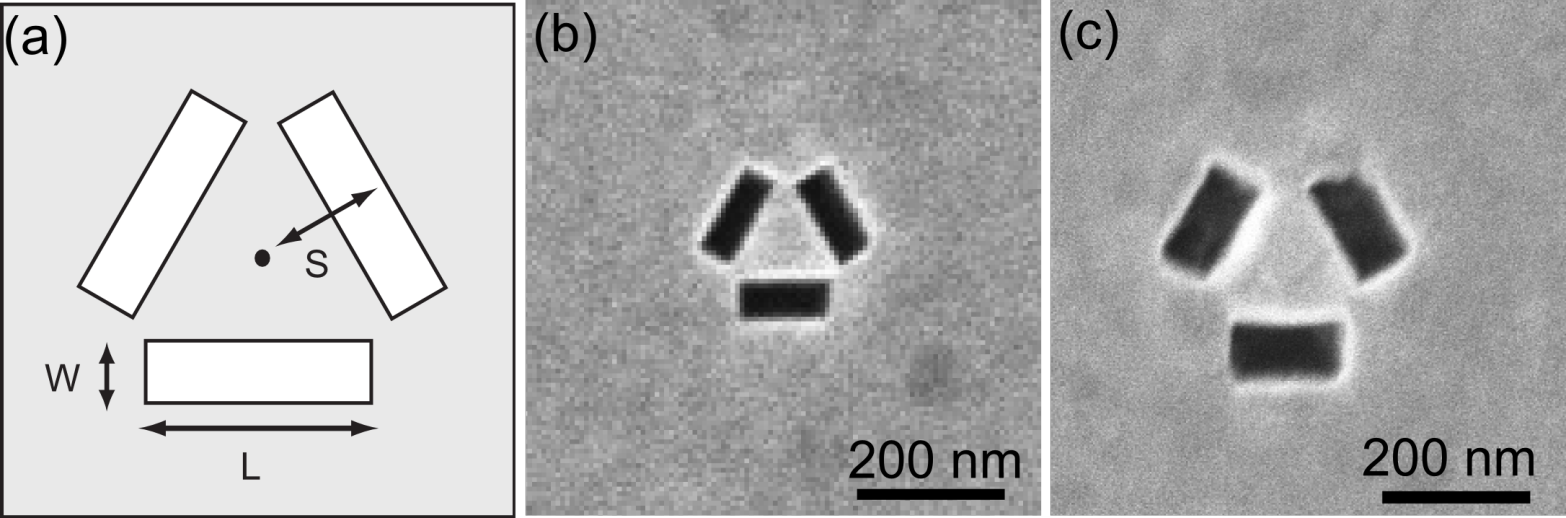
Scanning electron micrographs aperture structures with dimensions defined in (a). Fabricated slot trimer structures with (b) *L* = 95 nm, *W* = 34 nm and *S* = 60 nm, and (c) *L* = 150 nm, *W* = 40 and *S* = 100 nm.

A gold film of nominally 100 nm thickness is deposited using electron beam evaporation (Intvac Nanochrome I) onto a high quality borosilicate glass slide with 5 nm of chromium as an adhesive layer. The rate of deposition of the gold was set to 0.3 Å/s. High quality azimuthal arrangements of three slot apertures with different transverse parameters were milled using a helium ion microscope (Nanofab Orion, Zeiss) operating at an accelerating voltage of 30 kV and a beam current of 0.1 to 100 pA. A Fibics NPVE pattern generator was used to control the milling parameters such as dose, beam step size and dwell time. Test writing was performed on a 100 nm thick Au film on a borosilicate glass substrate. Initial exposures indicated a dose of 15 nC/cm^2^ as the optimal initial setting for the ion beam with a 1 µs dwell time and 50% beam overlap. The optimised ion beam current selected for milling was 1.5–2.4 pA, producing the highest quality apertures which typically required approximately 15 minutes of mill time. Slot trimers composed of slots of length *L* and width *W* arranged azimuthally in a triangle with a distance from the centre of each slot to the centroid of the configuration of *S* were fabricated. Scanning electron micrographs of the apertures investigated here are shown in Figure 1 along with a schematic illustrating the relevant parameters.

CL results were obtained using a scanning electron microscope (FEI NOVA Nano SEM 450) fitted with a Delmic SPARC CL system comprising an aluminium parabolic mirror with a moving stage to position the sample at the focus of the mirror [59]. A hole of 600 μm diameter is located in the mirror just above the focal point through which the electron beam passes and is then incident on the sample. A CL signal is emitted as the electron beam of 30 keV interacts with the specimen located at the focal point of the parabolic mirror. The optical CL signal reflected from the parabolic mirror is coupled into a 600 μm core diameter multimode optical fibre via an achromatic mirror. The fibre is connected to a spectrometer (PI Acton SP2300i) for CL spectral analysis. The schematic and details of the experimental CL set up can be found in various papers [60–61]. The background spectrum obtained from an adjacent, unpatterned region of the gold film is subtracted from all data and the result normalized by the system response. The system response function was found by obtaining the spectrum from an unpatterned region of gold film and normalizing this to the theoretical result for gold.

## Results

**Figure 2:**
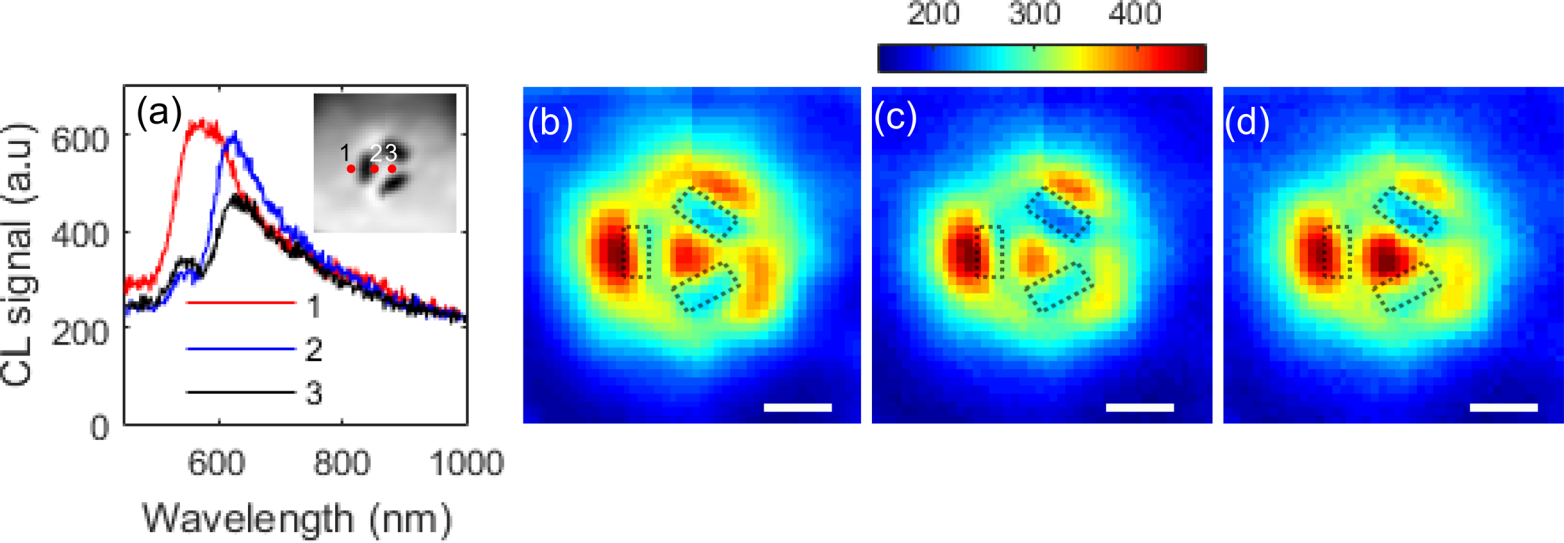
(a) CL spectra obtained from trimer slot structures with *L* = 95 nm, *W* = 40 nm and *S* = 60 nm obtained at different positions shown in the inset. The spatial dependence of CL emission in spectral bands (b) 550–560 nm, (c) 595–605 nm and (d) 610–620 nm are also shown. The scale bar in (b–d) is 100 nm.

Two different trimeric ensembles of slot apertures shown in Figure 1b and Figure 1c were investigated. In addition to cavity resonances associated with the aperture, this structure may also possess plasmonic resonances in the approximately triangular region between the apertures. We first consider a trimer consisting of slots with nominal length 100 nm, width 40 nm and with a separation of 60 nm as shown in Figure 1b. In the CL spectra shown in Figure 2a, two maxima are apparent when the electron beam is incident inside the region defined by the slots, but only a single, broad peak can be seen when the electron beam is incident just outside one of the slots.

If we examine the spatial maps of the CL emission in the wavelength ranges (Figure 2b) 550–560 nm and (Figure 2c) 595–605 nm, we see that the strongest signal occurs when the beam is incident just outside the edges of the slots, in contrast to the map centred on 615 nm (Figure 2d) where the strongest emission arises when the beam is centrally incident. Asymmetries apparent in Figure 2 arise from defects introduced during the fabrication process and there is discontinuity in the data resulting from a glitch in the electron beam scan that can be discerned in the concurrent SEM image.

**Figure 3:**
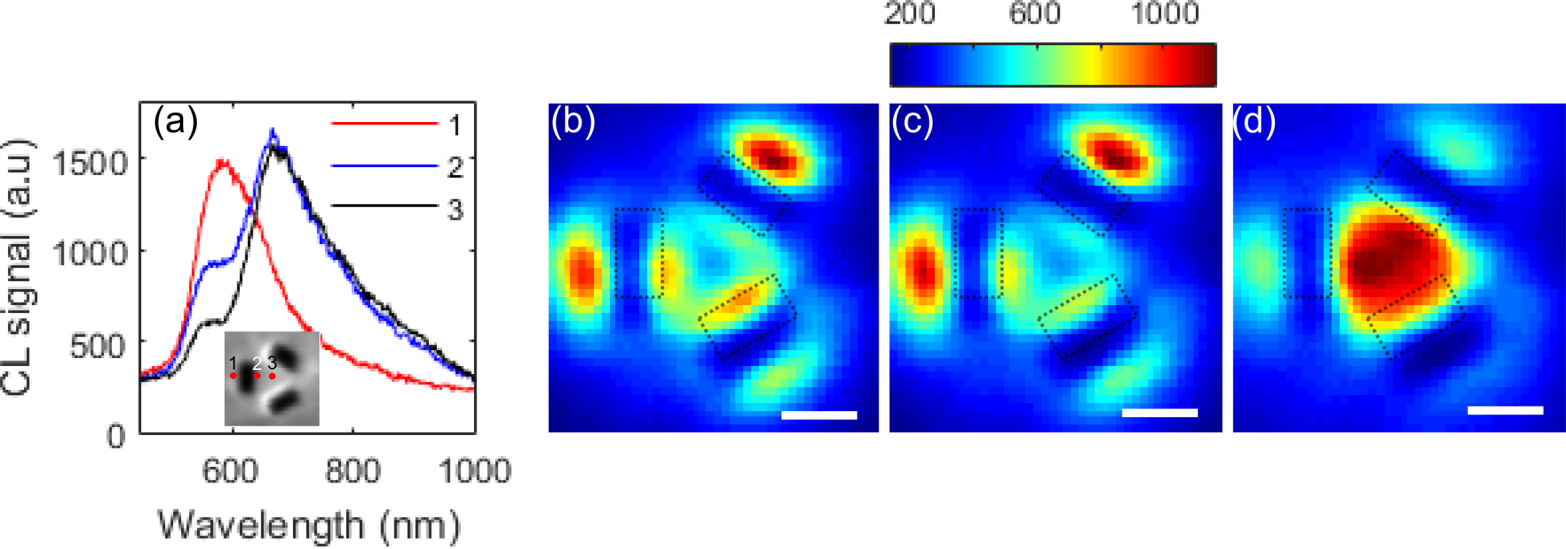
(a) CL spectrum from trimer slot structures with slot length 150 nm, width 65 nm and separation 100 nm obtained at different positions shown in the inset. Spatial variation of CL emission at wavelength bands (b) 550–560 nm, (c) 575–585 nm and (d) 665–675 nm. The scale bar in (b–d) is 100 nm.

The results from another structure with a length of 150 nm, width 65 nm and separation 100 nm with SEM shown in Figure 1c is shown in Figure 3 with spectra shown in Figure 3a along with spatial maps of emission in bands from 550–560 nm (Figure 3b), 575–585 nm (Figure 3c) and 665–675 nm (Figure 3d). Again, it is apparent that the CL spectrum obtained depends on the excitation point and that the emission into different wavelength bands depends on the spatial location. At shorter wavelengths, we see that excitation just outside the slots produces the strongest CL emission, but when electrons are incident in the central region, the peak in emission is red-shifted.

There are clear similarities between the CL responses from the two structures. Both appear to have two peaks in the CL spectrum when the electrons are incident on the centre of the ensemble. In both cases, the shorter wavelength resonance is located around 550 nm and appears to dominate when electrons are incident on position (1), that is, on the outer edge of one of the slots. The longer wavelength peak, however, is more strongly dependent on the geometry and is red-shifted for the larger structure.

## Simulations

The finite element method implemented in COMSOL Multiphysics (v 5.3) was used to gain insight into the experimentally obtained CL results shown in Figure 2 and Figure 3. In the model, a vertically oriented electric dipole placed at a height of 30 nm above the surface of the film [62] excites different resonances of the structure, leading to spectral variations in the back-emitted radiation and transmission through the apertures that depend on the transverse location of the dipole. A second order scattering boundary condition is used and no backscattering from the boundary into the modelled region was apparent. The far-field spectrum (which excludes evanescent contributions) radiated into a range of angles corresponding to a numerical aperture of 0.95 above the surface of the film is calculated by integrating |*E*|^2^ over the surface of the sphere subtended by this range of angles. Similarly, the far-field transmission through the apertures into a NA 0.95 cone is also calculated.

**Figure 4:**
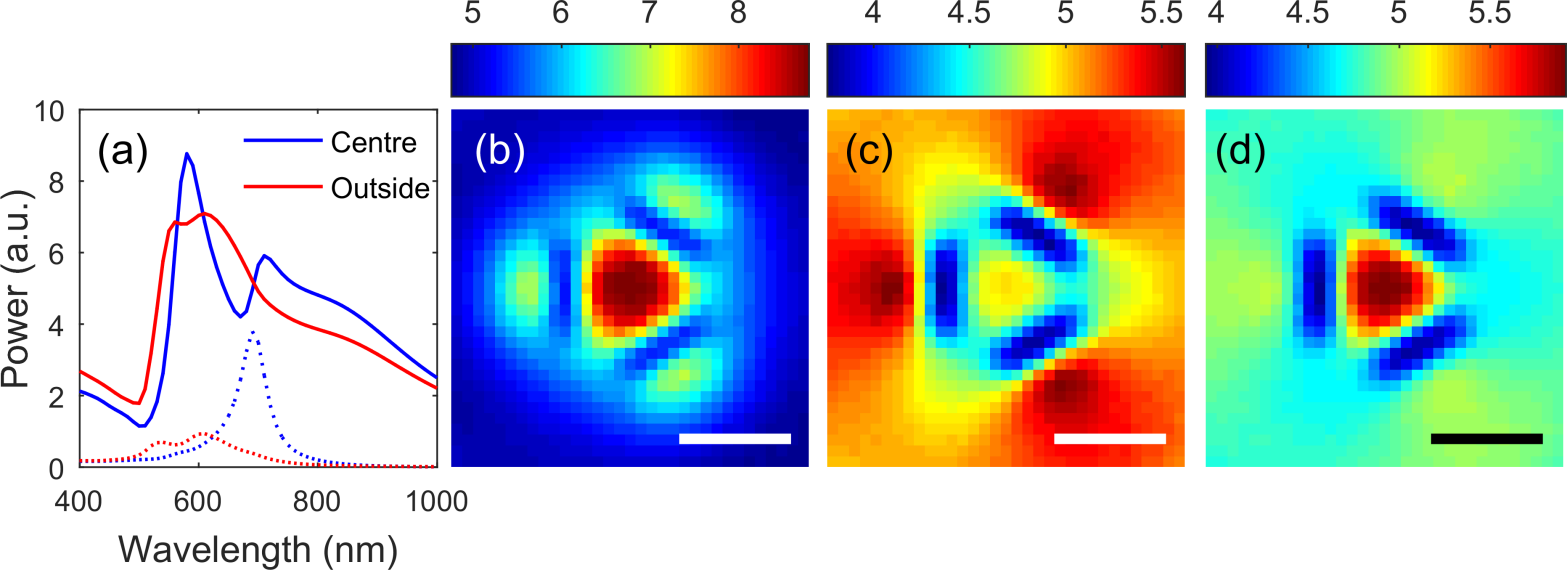
Simulated power radiated by a vertically oriented point dipole located 30 nm above a 100 nm thick gold film with three slots arranged in a triangular arrangement with length 100 nm and width 40 nm and separation of 60 nm. The spectra reflected from (solid curves) and transmitted through (dashed curves) the surface for dipoles located above the centre of the configuration and 5 nm outside the outer edge of one of the slots. The reflected power as a function of transverse dipole position is shown at wavelengths of (b) 580 nm, (c) 690 nm and (d) 710 nm. Scale bar is 100 nm.

Results for trimers with slots of length 100 nm and width 40 nm arranged with a separation of 60 nm are shown in Figure 4 corresponding approximately to the structure of Figure 1b with CL results shown in Figure 2. We plot both the back-emitted radiation toward the side of the gold film on which the dipole is located as well as the power transmitted through the aperture. Two distinct peaks in the back-emission spectra can be discerned as is the case with Figure 2a. For a centrally located dipole, the transmission through the aperture has a distinct maximum centred on 690 nm. As the dipole is moved to the outer edge of the ensemble, the transmission through the structure decreases and a broad peak, possibly associated with two resonances, located near 580 nm can be seen. This is consistent with the shorter wavelength maximum seen in the experimentally obtained CL spectrum of Figure 2a. This suggests that the longer wavelength feature in the back-emission is associated with strong coupling to the aperture. Looking at the spatial dependence of the backward emission at wavelengths of 580 nm (Figure 4b), 690 nm (Figure 4c) and 710 nm (Figure 4d), we can see that at the shorter wavelength, the backward emission at 580 nm is strongest when the dipole is centrally located, whereas at 690 nm (corresponding to the peak in the transmission spectrum if Figure 4a), we see strongest backward emission when the dipole is located over the outside edges of the slots. At slightly longer wavelengths (Figure 4d), however, we see a decrease in transmission and the sensitivity of the upward radiated power to position confined to the central region defined by the slots.

**Figure 5:**
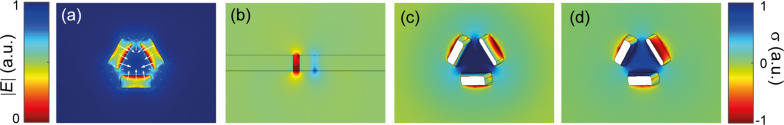
Electric field inside a trimer of rectangular slots of length 100 nm and width 40 nm in a gold film of thickness 100 nm at a wavelength of 690 nm excited by an electric dipole located 30 nm above the upper surface of the film and centred above the structure. The magnitude of the electric field (arrows showing direction) a distance of 5 nm above the lower surface of the gold film is shown in (a), while (b) shows the vertical component of the electric field in a vertical plane with reference to the geometry shown in (a). The (instantaneous) surface charge density on the metal boundary at a wavelength of (c) 580 nm and (d) 690 nm is also shown.

We can gain further insight by examining the electric field produced inside the cavities on resonance when excited by a centred dipole and the accompanying surface charge on the dielectric–metal or air–dielectric boundary (Figure 5). The white regions observable in the surface charge distributions correspond to the air-filled holes. Figure 5a shows the magnitude of the electric field with the arrows showing the direction of the electric field at a wavelength of 690 nm in a plane just above the lower surface of the gold film.

The dominant lowest order modes of this cavity ensemble consist of nearly degenerate “radial” and “dipole” modes. The dark radial mode has the electric field in each cavity directed radially from the centroid of the ensemble and has zero net dipole moment. The degenerate dipole modes, on the other hand, have orthogonally directed net dipole moments, but, as is the case with the radial mode, the electric field in each slot is directed normal to the long axis of slots, but each slot is not equally excited. An examination of the dispersion relations of a waveguide consisting of these three slots (see Supporting Information File 1) indicates that these radial and dipole modes have an effective “cut-off” at around 600 nm and a zeroth order Fabry–Pérot would be expected at a slightly longer wavelength in an aperture in a metal film with the same geometry. It is apparent from Figure 5a that the mode excited with a centred dipole has radial symmetry. Figure 5b shows that at 690 nm, where there is a maximum in transmission, the electric field penetrates the cavity. This is accompanied by a substantial induced surface charge on the interior walls of the cavity (Figure 5d) and the relative uniformity of the field and surface charge within the cavity as a function of depth (Figure 5b) suggests that this is a zeroth order Fabry–Pérot resonance of a cavity mode. The electric field (not shown) and induced surface charge at a wavelength of 580 nm (Figure 5c) corresponding to the shorter wavelength maximum in the back-emission spectrum are consistent with only weak penetration into the cavity and the resonance being associated with a resonance of surface plasmon polaritons (SPPs) of the approximately triangular region on the surface of the film defined by the apertures. Although a mode with radially symmetry is apparent, it was difficult to identify the dipole mode, even with off centre excitation.

Comparing the results of these simulations with the CL measurements of Figure 2 suggests that the longer wavelength resonance is associated with coupling into the cavity, which is consistent with the observation that the spectral location is dependent on geometry. The shorter wavelength resonance, on the other hand, is only weakly dependent on the aperture geometry and is likely to be more strongly related to the surface geometry defined by the inner edges of the apertures.

**Figure 6:**
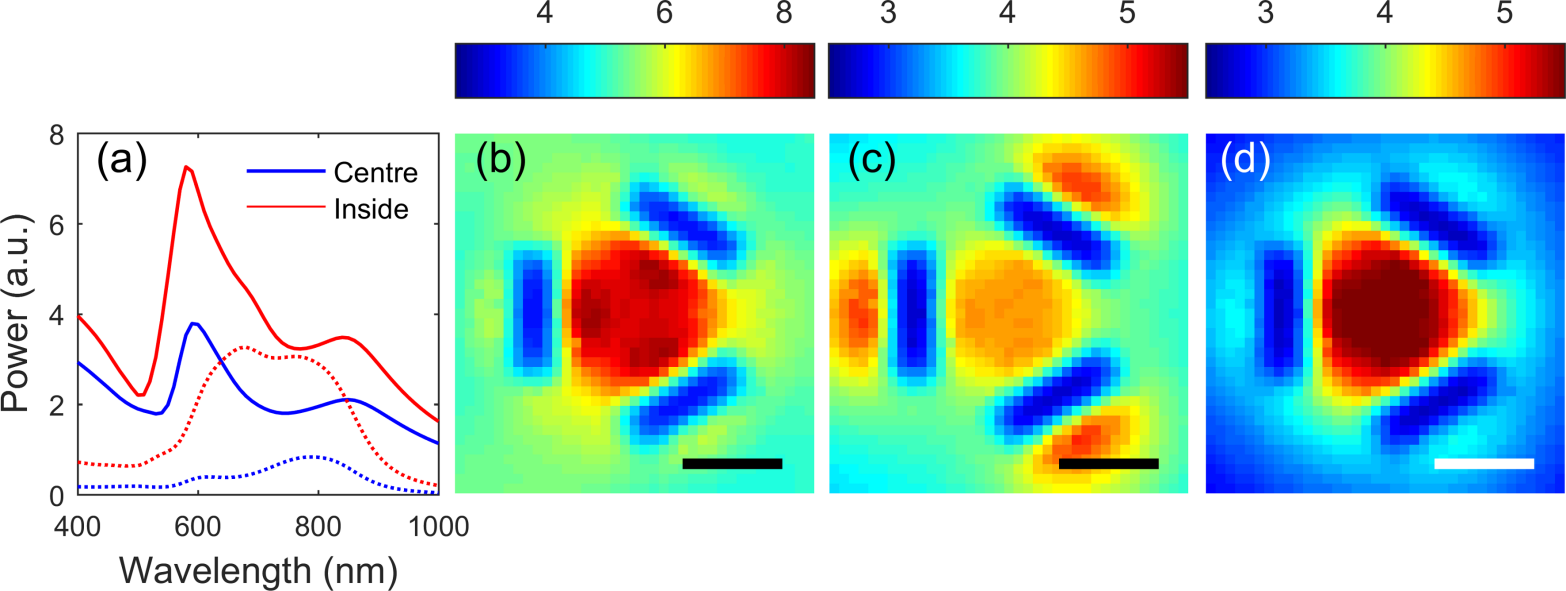
Simulated power radiated by a vertically oriented point dipole located 30 nm above a 100 nm thick gold film with three slots arranged in a triangular arrangement with length 150 nm and width 65 nm and separation of 100 nm. The spectra reflected from (solid curves) and transmitted through (dashed curves) the surface for dipoles located above the centre of the configuration and 5 nm outside the inner edge of one of the slots. The reflected power as a function of transverse dipole position is shown at wavelengths of (b) 600 nm, (c) 790 nm and (d) 860 nm. Scale bar is 100 nm.

The results of simulations of the structure with larger slots (length 150 nm, width 65 nm and separation 100 nm) corresponding approximately to the structure discussed in Figure 1c and the data of Figure 3 are shown in Figure 6. If we look at the emission spectrum (Figure 6a) obtained when the dipole is centrally located above the centre of the ensemble, we again see two distinct peaks in the back-radiated spectrum. Furthermore, two distinct, but close, resonances appear in the transmission spectrum. Moving the dipole to a location sitting above a point close to the inner edge of one of the slots produces both stronger back-radiation and transmission through the apertures. Comparing the spectra to those of the ensemble investigated in Figure 4, we see that the shorter wavelength resonance remains at approximately 600 nm, but the longer wavelength back-emission maximum has red-shifted to around 860 nm. Furthermore, the increased separation of the slots is accompanied by clear evidence for the excitation of a higher order SPP resonance on the gold–air boundary in the region between the slots (Figure 6b) at 600 m with a characteristic modal sensitivity to dipole position. At 790 nm (Figure 6c), corresponding to the longer wavelength maximum in the transmission spectra, we see a spatial dependence of emission with strong enhancement near the edges of the apertures consistent with the excitation of cavity modes. At a wavelength of 860 nm (Figure 6d) we see strong emission over the central region suggesting that the excitation of SPPs dominates the emission process.

**Figure 7:**
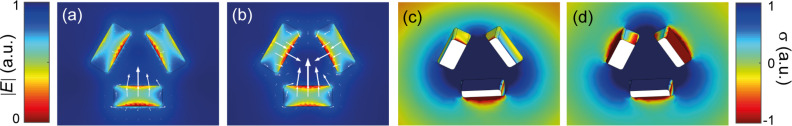
Electric field inside a trimer of rectangular slots of length 150 nm and width 65 nm separated by a distance of 100 nm in a gold film of thickness 100 nm excited by an electric dipole located 30 nm above a point on the film 5 nm inside the inner edge of the lower slot. The magnitude of the electric field (direction shown with arrows) in a plane a distance of 5 nm above the lower surface of the gold film at wavelengths of (a) 680 nm and (b) 800 nm. The (instantaneous) surface charge densities on the metal–dielectric boundary at wavelengths of (c) 600 nm and (d) 800 nm are also shown.

Examples of the electric field in the vicinity of the trimer when excited with an electric dipole sitting close to the inner edge of one of the slots are shown in Figure 7. The choice of dipole location was informed by the strong transmission through the structure for a dipole at this location as shown in Figure 6a and the fields are plotted at wavelengths of 680 nm and 800 nm, corresponding to peaks in the transmission (rather than the back-emission) spectrum. If we look at the electric field in a plane 5 nm above the bottom surface of the gold film at 680 nm (Figure 7a) and the surface charge (Figure 7c), we see that there is a net electric dipole moment associated with this mode. On the other hand, the electric field in the same plane at 800 nm (Figure 7b) has a radial symmetry indicating that this is the dark radial mode. This suggests that the degeneracy of the dipole and radial modes of the slot structure have been broken due to coupling between the apertures. This is consistent with the dispersion relation for the corresponding waveguide modes (see Supporting Information File 1) indicating a zeroth order Fabry–Pérot resonance for the radial mode at a longer wavelength than for the dipole mode. This effect is, however, too subtle to be seen in the CL measurements.

## Discussion

It is apparent that irradiation with an electron beam excites several resonances of these slot aperture structures. Although quantitative agreement between the CL results and simulations looking at radiation by a point electric dipole is weak, it appears that there are two distinct, but potentially coupled, processes occurring. Firstly, the electrons can excite SPPs on the gold–air interface and the regions defined by the boundaries of the apertures will support various resonances that depend on the geometry of that region. Secondly, modes of the cavities can be excited that transport energy through the aperture. The latter depend strongly on the geometry of the aperture configuration. The FEM simulations suggest that both these types of resonances are being excited in our experiments and producing measurable CL emission.

In the case of the rectangular aperture trimers investigated here, the cavities are only weakly coupled, but some evidence can be seen in the excitation of both the dipole and radial modes in the simulations. Angle-resolved CL measurements could be used to obtain further information to assist in the identification of different modes since the far-field radiation patterns are quite different for radial and dipolar modes. Ongoing work is aimed at exploring different geometries in gold films of varying thickness. This will provide an opportunity to elucidate the role played by the thickness of the film, if significant, in various resonances. Although challenging, studying the optical transmission through these apertures may similarly yield interesting results.

## Conclusion

We have investigated ensembles of rectangular slots in a gold film on a glass substrate and using CL we have shown that various modes of the system can be excited. Modelling results show that these resonances may be accompanied by transmission through the aperture, suggesting that cavity resonances of the apertures are being excited. There are however, spectral features that are not accompanied by any significant transmission through the aperture, suggesting that these are more closely associated with resonances arising from the surface structure on the air–metal boundary and have more in common with previous studies on metallic nanoantennas. The existence of these two different modes within this structure raises the prospect of designing aperture structures where the transverse geometry could be tailored to facilitate coupling between cavity and plasmonic resonances. This could underpin novel approaches to controlling the emission of molecules and quantum dots for applications ranging from sensing through to new types of displays.

## Supporting Information

File 1 Additional figures.
Background material relating to the modes of rod and aperture ensembles.
