# Supplementary material for: Cathodoluminescence as a probe of the optical properties of resonant apertures in a metallic film
Source: Beilstein J Nanotechnol. 2018 May 18;9:1491–500. doi: 10.3762/bjnano.9.140 (PMC6009612; doi:10.3762/bjnano.9.140)
Supplement: File 1 — Additional figures. Background material relating to the modes of rod and aperture ensembles. [file Beilstein_J_Nanotechnol-09-1491-s001.pdf]

Supporting Information

for

Cathodoluminescence as a probe of the optical properties of resonant apertures in a metallic film

Kalpana Singh[1*], Evgeniy Panchenko[1], Babak Nasr[2,3,4], Amelia Liu[5], Lukas Wesemann[1], Timothy J. Davis[1*] and Ann Roberts[1*]

Address: [1]School of Physics, University of Melbourne, VIC 3010 Australia, [2]Centre for Neural Engineering, The University of Melbourne, VIC 3010, Australia, [3]Department of Electrical and Electronic Engineering, The University of Melbourne, VIC 3010, Australia, [4]Australian Research Council Centre of Excellence for Integrative Brain Function, The University of Melbourne, VIC 3010, Australia, and [5] Monash Centre for Electron Microscopy and School of Physics and Astronomy**,** Monash University, Clayton, VIC 3800 Australia

* Corresponding author

Email: Timothy J. Davis - timd@unimelb.edu.au; Kalpana Singh - ksingh1@student.unimelb.edu.au; Ann Roberts - ann.roberts@unimelb.edu.au

Additional Figures

Modes of a nanorod trimer

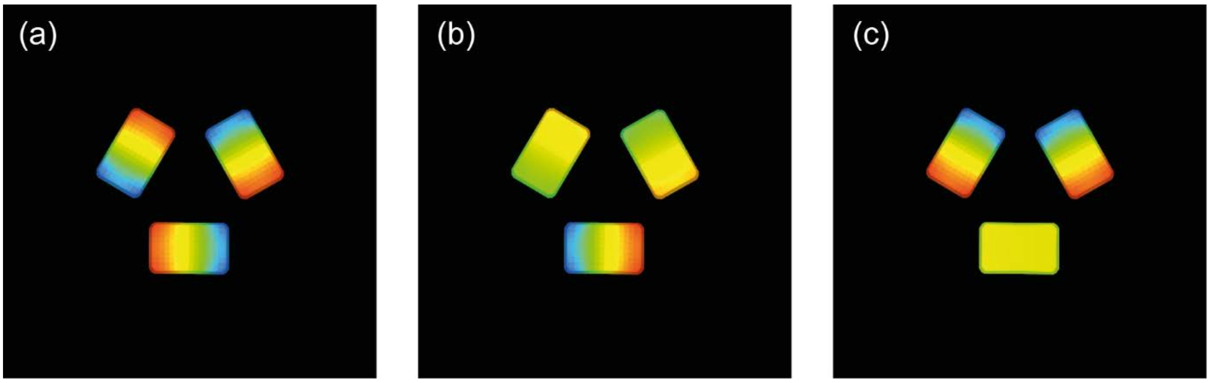

Figure S1: Surface charge distributions on azimuthal arrangements of rods complementary to the slot ensembles considered here. The lowest energy mode (a) is dark and has the dipole moments on each rod arranged azimuthally. The next highest energy modes are bright, degenerate dipole modes with net horizontal (b) and vertical (c) dipole moments.

Waveguide modes for azimuthal trimer aperture in gold film

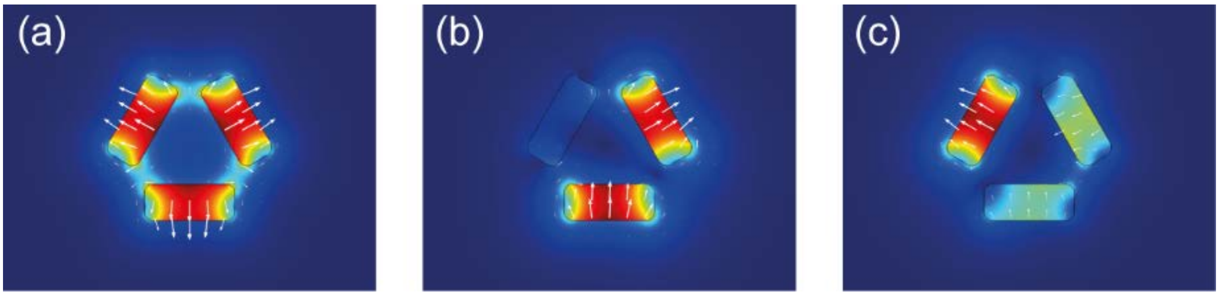

Figure S2: Magnitude of the electric field for the three lowest-order modes propagating in a trimer slot waveguide with slot length and width 100 nm and 40 nm respectively and with a separation (distance from centre of slot to centroid) of 60 nm. Modes solved using the Finite Element Method (COMSOL Multiphysics v5.3) using optical constants of gold taken from Johnson and Christie. Mode 1 (a) has a radial

symmetry with zero net electric dipole moment. This dark mode exhibits a radial symmetry and is referred to as the ‘radial’ mode.. Mode 2 describes degenerate ‘dipole’ modes (b) and (c) with net dipole moments directed at 60^0 (b) and 30^0 (c) to the horizontal as shown in the figures.

Dispersion curves for a rectangular and azimuthal trimer waveguides

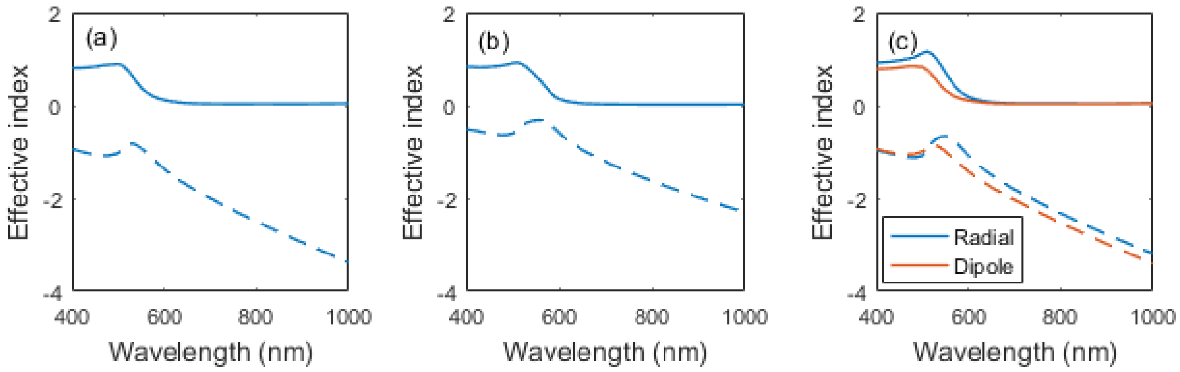

Figure S3: The effective index as a function of free-space wavelength for air-filled waveguides in gold. Modes solved using the Finite Element Method (COMSOL Multiphysics v5.3) with optical constants of gold taken from Johnson & Christy. Plots (a) and (b) are for single rectangular waveguides with length and width (a) 100 nm and 40 nm, and (b) 150 nm and 65 nm, respectively. The plot in (c) shows the effective index of radial and dipole modes the trimer shown in Figure S2. In all plots, the real part of the effective index is shown as a solid line and the imaginary part as a dashed line.
